# LncRNA-LALR1 upregulates small nucleolar RNA SNORD72 to promote growth and invasion of hepatocellular carcinoma

**DOI:** 10.18632/aging.102907

**Published:** 2020-03-11

**Authors:** Lin-Hong Mao, Si-Yuan Chen, Xiao-Qin Li, Feng Xu, Jing Lei, Qing-Liang Wang, Li-Yang Luo, Hai-Yan Cao, Xin Ge, Tao Ran, Xue Li, Min Zou, Zhi-Hang Zhou, Xiao-Ling Wu, Song He

**Affiliations:** 1 Department of Gastroenterology, The Second Affiliated Hospital of Chongqing Medical University, Chongqing, China; 2 Department of Pathology, The Second Affiliated Hospital of Chongqing Medical University, Chongqing, China

**Keywords:** lncRNA-LALR1, SNORD72, ID2, HCC, mRNA stability

## Abstract

Hepatocellular carcinoma (HCC) is one of the most prevalent cancers and currently the second leading cause of cancer-related mortality worldwide. One recent study reported that lncRNA-LALR1 promotes liver regeneration, the role and underlying mechanisms of lncRNA-LALR1 in HCC remain largely unknown. In this study, we demonstrated that lncRNA-LALR1 was significantly upregulated in HCC tissues compared with adjacent tissues and high expression of lncRNA-LALR1 was associated with advanced TNM stage, poor differentiation, and distant metastasis. RNA Fluorescence in situ hybridization analysis showed lncRNA-LALR1 was expressed not only in cytoplasm but also in nucleolus. Knockdown of lncRNA-LALR1 obviously inhibited HCC cells growth and invasion *in vivo* and *in vitro*. Besides, transcriptomic analysis and subsequent confirmation revealed that lncRNA-LALR1 upregulated small nucleolar RNA SNORD72 via binding with SNORD72 and stabilized ID2 mRNA. SNORD72 was overexpressed in HCC tissues and enhanced HCC cells proliferation, colony formation and invasion. Overexpression of SNORD72 could also stabilize ID2 mRNA and rescue the inhibitory effect of silencing lncRNA-LALR1. In conclusion, lncRNA-LALR1 is highly expressed in HCC and promotes tumor growth and invasion by upregulating SNORD72 to stabilize ID2 mRNA, implying that lncRNA-LALR1 might be a novel target for intervention of HCC.

## INTRODUCTION

Hepatocellular carcinoma (HCC) is the second most common reason for cancer-related death worldwide, especially in South-Eastern Asia and Africa, causing nearly 745,000 deaths every year [1]. Although the diagnosis and treatment technology of HCC have been improved in recent decades, the survival time of HCC patients remains one of the shortest among all cancers. This fact is largely due to its complicated pathogenesis [2]. It is therefore urgently needed to reveal further molecular mechanisms of HCC for developing new diagnostic and therapeutic strategies to improve the prognosis of HCC patients. Long non-coding RNA (lncRNA) is a class of noncoding transcripts, which are larger than 200 nucleotides. Recent studies have revealed lncRNAs, such as MEG-3, MALAT1, HULC, HOTAIR, and H19, play pivotal role in HCC progression [3]. They modulate several biological processes including cell proliferation, apoptosis, epithelial-mesenchymal transition, invasion, metastasis and autophagy [3]. LncRNAs can bind with DNA, RNA or proteins to regulate the expression and function of target molecules at transcriptional and post-transcriptional levels. One previous study reported that lncRNA-LALR1 promoted hepatocytes proliferation by inducing cell cycle during mouse liver regeneration [4], but its role in HCC remained largely unknown.

Small nucleolar RNAs (snoRNAs) are abundantly expressed non-coding RNAs with 60–300 nucleotides in length. They are mainly located in nucleolus and divided into C/D box snoRNAs (SNORDs) and H/ACA box snoRNAs (SNORAs). Classically, the SNORDs mediate the O-methylation of the target molecule, while the SNORAs mediate the pseudourine of the target molecule [5]. But recent studies revealed that nearly half of SNORDs might have functions other than methylation, as sequence alignment showed that they did not have methylation targets [6]. Gong Jing and colleagues analyzed the expression of snoRNAs in 31 kinds of tumors including HCC, breast cancer, colorectal cancer and lung cancer and found that snoRNAs were highly expressed in tumor tissues compared with paracancerous tissues [5]. Besides, the expression of SNORDs was significantly higher than SNORAs [5]. Few studies have reported that some SNORD family members acted to promote or suppress tumor progression. For example, SNORD78 was highly expressed in non-small cell lung cancer and promoted cell proliferation, invasion and stemness maintenance [7]. While SNORD113-1 could inhibit HCC cells proliferation and its expression was positively correlated with favorable prognosis of HCC patients [8]. However, the role of other SNORD family members in HCC remained largely unknown.

Inhibitors of DNA binding and cell differentiation (ID) proteins belong to the basic helix-loop-helix (bHLH) transcription factor family, including ID1-ID4. They can affect cell growth and differentiation by inhibiting the binding of transcription factors to DNA [9]. Previous study reported that ID1/2/3 was highly expressed in multiple tumors including HCC, while ID4 was lowly expressed in breast cancer, prostate cancer [10]. Among them, ID2 could enhance glioma and colorectal cancer formation [11]. Besides, ID2 was highly expressed in multiple cancers, including breast cancer, bladder cancer, pancreatic cancer, colorectal cancer and head and neck cancer [12]. Recent studies had shown that ID2 could not only promote the proliferation and inhibit apoptosis of HCC cells [13, 14], but also induce invasion and metastasis of HCC cells [15]. However, the regulatory mechanism of ID2 expression remains elusive.

In the present study, we found that lncRNA-LALR1 was highly expressed in HCC tissues compared with adjacent tissues and high expression of lncRNA-LALR1 was associated with advanced TNM stage, poor differentiation, and distant metastasis. Knockdown of lncRNA-LALR1 significantly inhibited growth and invasion of HCC cells *in vivo* and *in vitro*. Besides, we demonstrated that lncRNA-LALR1 could directly bind to SNORD72 and stabilize ID2 mRNA. Moreover, SNORD72 was overexpressed in HCC tissues and promoted the proliferation, colony formation and invasion of HCC cells. Overexpression of SNORD72 could also increase ID2 mRNA stability and rescue the inhibitory effect of silencing lncRNA-LALR1. In conclusion, lncRNA-LALR1 could promote growth and invasion of HCC cells via SNORD72/ID2 axis, implying that lncRNA-LALR1 might be a novel diagnostic or therapeutic target for HCC.

## RESULTS

### Overexpression of lncRNA-LALR1 correlates with advanced TNM stage, poor differentiation, and distant metastasis of HCC

**Figure 1:**
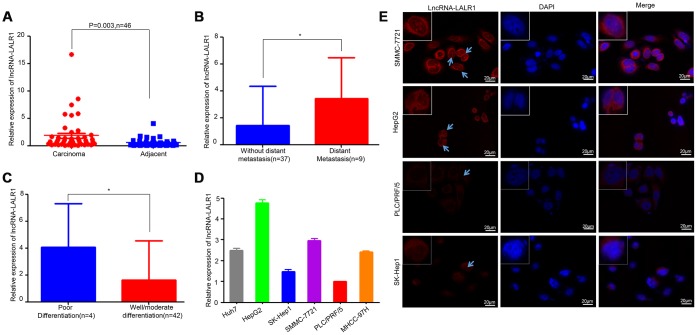
**LncRNA-LALR1 is highly expressed in HCC samples.** (**A**) qRT-PCR assay showing the expression of lncRNA-LALR1 in HCC tissues and matched non-tumor tissues. (**B**) The expression of lncRNA-LALR1 in tumor samples from HCC patients with distant metastasis. (**C**) The expression of lncRNA-LALR1 in tumor samples from HCC patients with poor differentiation (**D**) qRT-PCR assay showing the expression of lncRNA-LALR1 in six HCC cell lines. (**E**) Fluorescence in situ hybridization (FISH) assay showing the localization of lncRNA-LALR1 in HCC cells. *: P < 0.05.

The expression of lncRNA-LALR1 was examined by qRT-PCR in 46 patients, including 36 males and 10 females. Results showed that lncRNA-LALR1 was upregulated in HCC tissues as compared with adjacent non-tumor tissues (Figure 1A). LncRNA-LALR1 was significantly highly expressed in tumor samples from HCC patients with distant metastasis (*P*=0.043) (Figure 1B) or poor differentiation (*P*=0.03) (Figure 1C). Chi-square test revealed that high lncRNA-LALR1 expression significantly correlated with advanced TNM stage (*P*=0.033), poor differentiation (*P*=0.048) and distant metastasis (*P*=0.004) (Table 1) in HCC. These results indicate lncRNA-LALR1 is associated with progression of HCC.

**Table 1:** Correlation between lncRNA-LALR1 expression and clinicopathological features in cancer tissues from 46 HCC patients.

**Factors**	**No. of patients (%)**	**LncRNA-LALR1^#^**		**χ2 value**	***P* value**
		**low(n=35)**	**high(n=11)**		
		**No. of patients (%)**	**No. of patients (%)**		
Gender				0.558	0.455
Male	36(78.3)	26(72.2)	10(27.8)		
Female	10(21.7)	9(90.0)	1(10.0)		
Age(years)				0	1
≤ 50	14(30.4)	11(78.6)	3(21.4)		
>50	32(69.6)	24(75.0)	8(25.0)		
Size(cm)				0.537	0.464
≤ 5	27(58.7)	19(70.4)	8(29.6)		
>5	19(41.3)	16(84.2)	3(15.8)		
HBsAg				0.287	0.592
Negative	8(17.4)	5(62.5)	3(37.5)		
Positive	38(82.6)	30(78.9)	8(21.1)		
AFP (ng/ml)				0.717	0.397
≤ 20	18(39.1)	12(66.7)	6(33.3)		
> 20	28(60.9)	23(82.1)	5(17.9)		
ALB (g/L)				0.157	1
≤ 35	3(6.5)	2(66.7)	1(33.3)		
>35	43(93.5)	33(76.7)	10(23.3)		
Liver cirrhosis				0	1
No	20(43.5)	15(75.0)	5(25.0)		
Yes	26(56.5)	20(76.9)	6(23.1)		
Tumor number				0.406	0.524
Single	32(69.6)	23(71.9)	9(28.1)		
Multiple	14(30.4)	12(85.7)	2(14.3)		
T stage				0.004	0.949
I+II	31(67.4)	23(74.2)	8(25.8)		
III+IV	15(32.6)	12(80.0)	3(20.0)		
N stage				1.647	0.199
Negative	34(73.9)	28(82.4)	6(17.6)		
Positive	12(26.1)	7(58.3)	5(41.7)		
TNM stage				8.073	**0.033**
I	12(26.1)	11(91.7)	1(8.3)		
II	6 (13.0)	5(83.3)	1(16.7)		
III	12(26.1)	11(91.7)	1(8.3)		
IV	16(34.8)	8(50.0)	8(50.0)		
Vascular invasion				0.323	0.57
Negative	37(80.4)	27(73.0)	10(27.0)		
Positive	9(19.6)	8(88.9)	1(11.1)		
Lymphnode metastasis				0.008	0.927
No	36(78.3)	28(77.8)	8(22.2)		
Yes	10(21.7)	7(70.0)	3(30.0)		
Distant metastasis				8.509	**0.004**
No	37(80.4)	32(86.5)	5(13.5)		
Yes	9(19.6)	3(33.3)	6(66.7)		
Differentiation				5.195	**0.048**
Well	7(15.2)	6(85.7)	1(14.3)		
Moderate	35(76.1)	28(80.0)	7(20.0)		
Poor	4(8.7)	1(25.0)	3(75.0)		

Chi-square test was used to evaluate the correlation between lncRNA-LALR1 expression and clinicopathological features. The bold values indicated that the *P* value was smaller than 0.05.

### The expression and location of lncRNA-LALR1 in HCC cells

To explore the functions of lncRNA-LALR1, we firstly examined the expression of lncRNA-LALR1 in HCC cells, including Huh7, HepG2, Sk-Hep1, SMMC-7721, PLC/PRF/5, and MHCC-97H cells. The qRT-PCR analysis revealed significantly higher lncRNA-LALR1 expression in HepG2 and SMMC-7721 cells than other HCC cells (Figure 1D). The expression and location of lncRNA-LALR1 was also investigated by RNA fluorescence in situ hybridization analysis (FISH). Consistently, FISH revealed that the expression of lncRNA-LALR1 in HepG2 and SMMC-7721 cells was stronger than other cells (Figure 1E). And the transcript of lncRNA-LALR1 was located not only in the cytoplasm, but also in the nucleus. These results demonstrate that lncRNA-LALR1 is expressed in both cytoplsm and nucleus in HCC cells.

### LncRNA-LALR1 promotes HCC progression in vitro and in vivo

**Figure 2:**
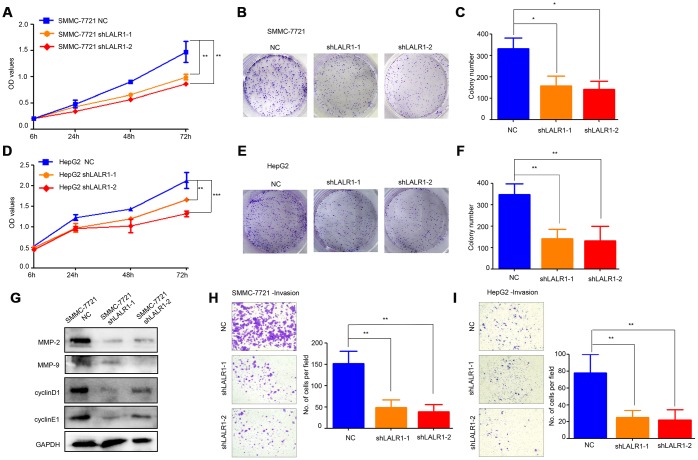
**Knock-down of lncRNA-LALR1 suppresses growth and invasion of HCC cells in vitro.** (**A**) CCK-8 assay showing knockdown of lncRNA-LALR1 decreases the proliferation ability compared with control group of SMMC-7721 cells. (**B**) Colony formation assay showing lncRNA-LALR1 knockdown reduces the growth ability compared with control group of SMMC-7721 cells. (**C**) Colony formation assay showing lncRNA-LALR1 knockdown reduces the growth ability compared with control group of SMMC-7721 cells. (**D**) CCK-8 assay showing knockdown of lncRNA-LALR1 decreases the proliferation ability compared with control group of HepG2 cells. (**E**) Colony formation assay showing lncRNA-LALR1 knockdown reduces the growth ability compared with control group of HepG2 cells. (**F**) Colony formation assay showing lncRNA-LALR1 knockdown reduces the growth ability compared with control group of HepG2 cells. (**G**) Western Blot assay showing knockdown of lncRNA-LALR1 downregulates cyclinD1, cyclinE1, MMP-2 and MMP-9. (**H**) Transwell invasion assay showing lncRNA-LALR1 silencing decreases the invasion ability compared with control group of SMMC-7721 cells. (**I**) Transwell invasion assay showing lncRNA-LALR1 silencing decreases the invasion ability compared with control group of HepG2 cells. *: P < 0.05; **: P < 0.01; ***: P < 0.001.

**Figure 3:**
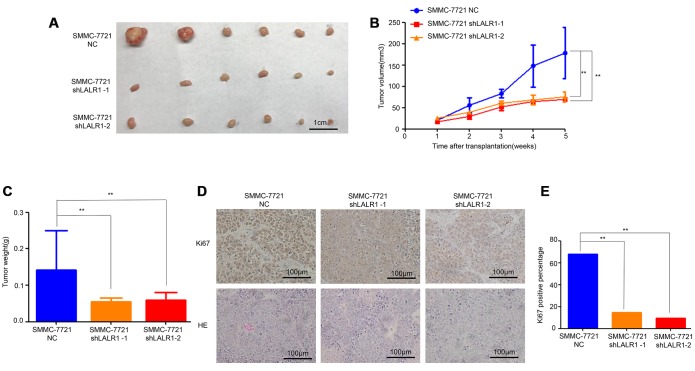
**Knock-down of lncRNA-LALR1 inhibits growth of HCC cells *in vivo*.** (**A**) The tumor volumes after knockdown of lncRNA-LALR1. (**B**) Knockdown of lncRNA-LALR1 decreases tumor volumes. (**C**) Knockdown of lncRNA-LALR1 decreases tumor weights. (**D**) Immunohistochemistry showing lncRNA-LALR1 silencing leads to a reduce of Ki67 protein levels. (**E**) LncRNA-LALR1 silencing reduces Ki67 protein levels. *: P < 0.05; **: P < 0.01; ***: P < 0.001.

HepG2 and SMMC-7721 cells, in which lncRNA-LALR1 was highly expressed, were transfected with lentivirus particles containing shRNA against lncRNA-LALR1 to investigate the biological functions of lncRNA-LALR1 (Supplementary Figure 1). We found that knockdown of lncRNA-LALR1 significantly inhibited the proliferation (*P*<0.01) (Figure 2A), and clonogenicity (*P*<0.05) (Figure 2B, 2C) of SMMC-7721 cells. Similar phenomena were also observed in HepG2 cells (*P*<0.001) (Figure 2D–2F). Knockdown of lncRNA-LALR1 did not affect cell cycle or apoptosis of HCC cells by flow cytometry (Supplementary Figure 2). However, cyclinD1, cyclinE1 were downregulated by knockdown of lncRNA-LALR1 (Figure 2G). Furthermore, we demonstrated that knockdown of lncRNA-LALR1 dramatically inhibited invasion of both SMMC-7721 (*P*<0.01) and HepG2 cells (*P*<0.01) (Figure 2H–2I). And the expression of MMP-2 and MMP-9 were decreased after lncRNA-LALR1 silencing (Figure 2G). Subcutaneous transplantation showed that knockdown of lncRNA-LALR1 also significantly decreased tumor volume (*P*<0.01) (Figure 3A, 3B) and weight (*P*<0.01) (Figure 3C). LncRNA-LALR1 silencing reduced the positivity of Ki67 protein (*P*<0.01), which reflects cell proliferation (Figure 3D–3E). All together, these results demonstrate that lncRNA-LALR1 promotes progression of HCC both *in vitro* and *in vivo*.

### Knockdown of lncRNA-LALR1 changes the expression of genes associated with nuclear processes

**Figure 4:**
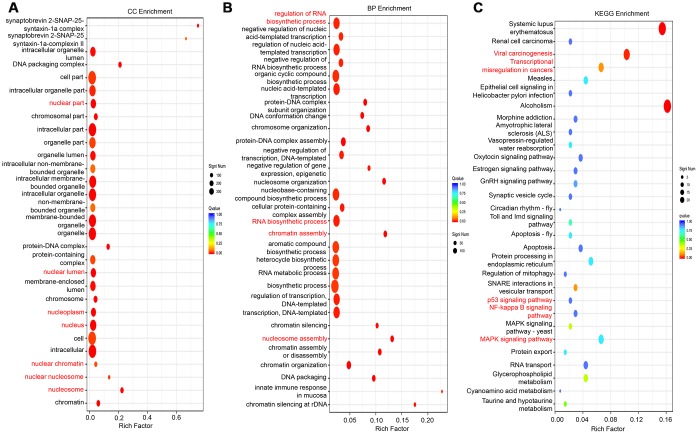
**GO and KEGG analysis of differentially expressed genes after knockdown of lncRNA-LALR1.** (**A**) Cellular component analysis of the differentially expressed genes. (**B**) Cellular process analysis of the differentially expressed genes. (**C**) KEGG signaling analysis of the differentially expressed genes.

RNA sequencing was applied to explore the gene expression profile changes after lncRNA-LALR1 silencing. The results showed that knockdown of lncRNA-LALR1 upregulated 540 genes and downregulated 145 genes (fold change>2, P<0.05) (Supplementary Figure 3A). GO analysis revealed that lncRNA-LALR1 was profoundly correlated with nuclear components and processes (Figure 4A–4B, Supplementary Figure 3B). Furthermore, KEGG analysis determined that lncRNA-LALR1 silencing significantly changed signaling pathways such as p53 signaling pathway, NF-κB signaling pathway and MAPK signaling pathway (Figure 4C). These results showed that lncRNA-LALR1 might promote the progression of HCC through regulating nuclear processes.

### LncRNA-LALR1 upregulates SNORD72 and ID2 in HCC cells

**Figure 5:**
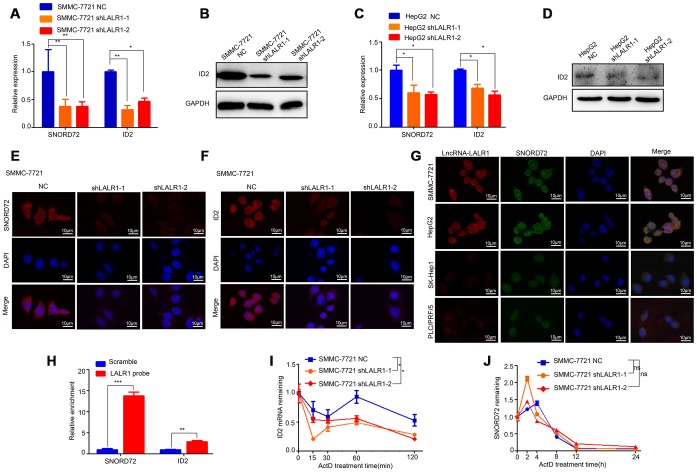
**LncRNA-LALR1 upregulates SNORD72 and ID2 in HCC cells.** (**A**) qRT-PCR showing the expression of SNORD72 and ID2 after lncRNA-LALR1 silencing in SMMC-7721 cells. (**B**) Western blot showing the expression of ID2 after lncRNA-LALR1 silencing in SMMC-7721 cells. (**C**) qRT-PCR showing the expression of SNORD72 and ID2 after knockdown of lncRNA-LALR1 in HepG2 cells. (**D**) Western blot showing the expression of ID2 after lncRNA-LALR1 silencing in HepG2 cells. (**E**) FISH assay showing the expression of SNORD72 after lncRNA-LALR1 silencing in SMMC-7721 cells. (**F**) FISH assay showing the expression of ID2 after lncRNA-LALR1 silencing in SMMC-7721 cells. (**G**) FISH showing lncRNA-LALR1 is co-localized with SNORD72 in HepG2, SMMC-7721, PLC/PRF/5, Sk-Hep1 cells. (**H**) RNA pulldown assay showing lncRNA-LALR1 interacts with SNORD72 and ID2 mRNA. (**I**) The effect of lncRNA-LALR1 on the mRNA stability of ID2. (**J**) The effect of lncRNA-LALR1 on the mRNA stability of SNORD72. *: P < 0.05; **: P < 0.01; ***: P < 0.001; ns: no significant.

The expressions of the top 16 differentially expressed genes were then verified by qRT-PCR. The results showed that knockdown of lncRNA-LALR1 significantly decreased SNORD72 (*P*<0.01) and ID2 expression (*P*<0.01) (Supplementary Figure 3C). We also found that knockdown of lncRNA-LALR1 lowered the expression of other snoRNAs (*P*<0.01) (Supplementary Figure 4). We verified that knockdown of lncRNA-LALR1 significantly decreased the expression of SNORD72 and ID2 in both SMMC-7721 (*P*<0.01) and HepG2 cells (*P*<0.05) (Figure 5A–5D). FISH assay also confirmed the results (Figure 5E–5F). Although it has been reported snoRNAs could shuttle between cytoplasm and nucleolus, the sub-cellular localization of SNORD72 has yet to be revealed. We found that SNORD72 was expressed in both cytoplasm and nucleolus (Figure 5G). Interestingly, we found that lncRNA-LALR1 co-localized with SNORD72 in HepG2, SMMC-7721, PLC/PRF/5, and SK-Hep1 cells (Figure 5G). RNA pulldown assay revealed that lncRNA-LALR1 could interact with SNORD72 (*P*<0.001) and ID2 mRNA (*P*<0.01) (Figure 5H). As lncRNAs could regulate the target molecules expression at post-transcriptional levels [16], we tested the effect of lncRNA-LALR1 on the mRNA stability of SNORD72 and ID2 mRNA in HCC cells. We found that knockdown of lncRNA-LALR1 significantly decreased the stability of ID2 mRNA (*P*<0.05) (Figure 5I) but not SNORD72 (*P*>0.05) (Figure 5J). Taken together, these results demonstrated that lncRNA-LALR1 unpregulates SNORD72 and stabilizes ID2 mRNA in HCC cells.

### SNORD72 promotes proliferation and invasion of HCC cells

**Figure 6:**
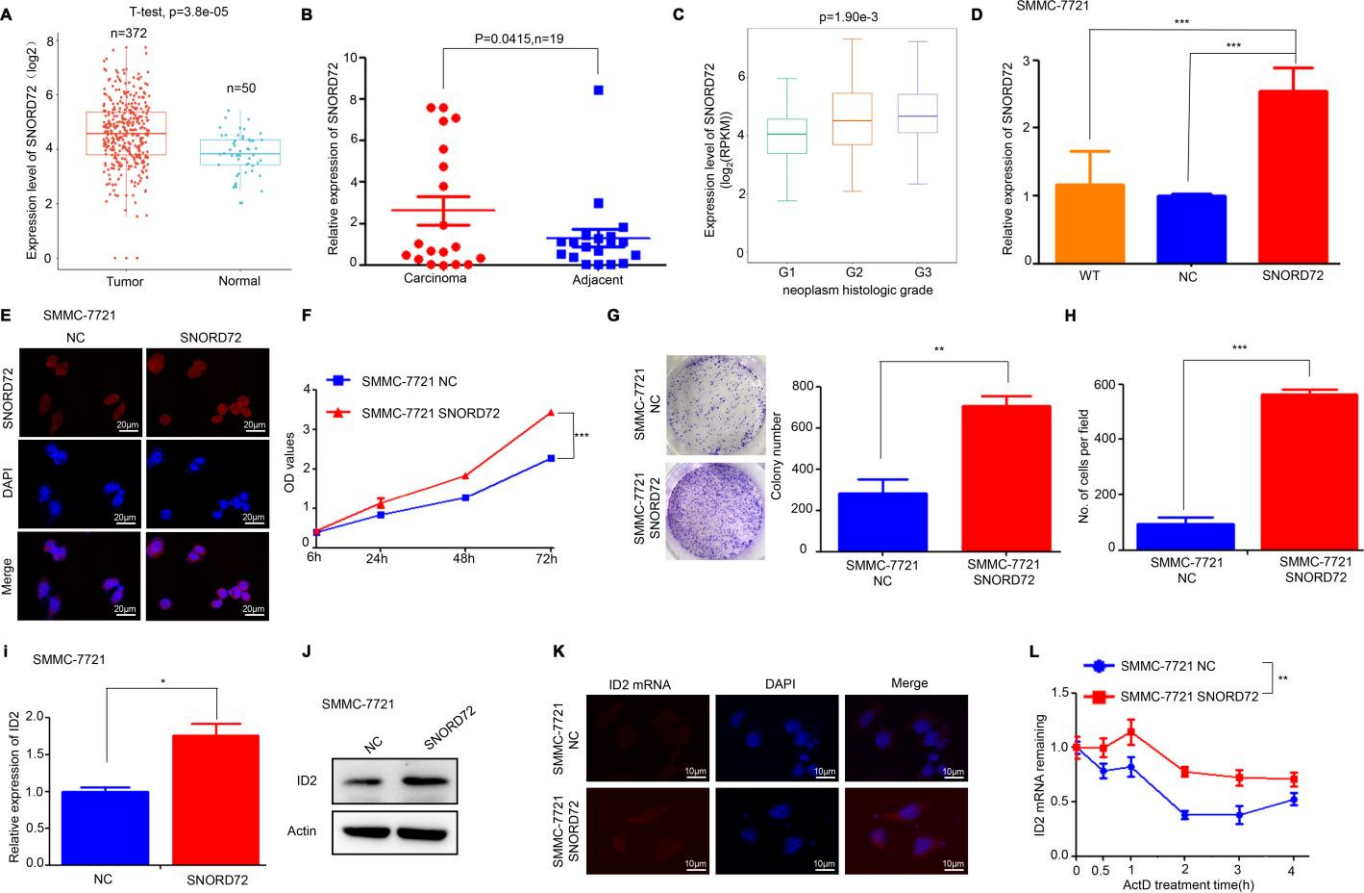
**SNORD72 is upregulated in HCC samples and promotes proliferation and invasion of HCC cells.** (**A**) Bioinformatic analysis showing the expression of SNORD72 in HCC tissues and adjacent non-tumor tissues. (**B**) qRT-PCR assay showing the expression of SNORD72 in HCC tissues and matched non-tumor tissues. (**C**) Bioinformatic analysis showing the relationship between SNORD72 expression and the differentiation degree. (**D**) qRT-PCR assay verifying the overexpression of SNORD72. (**E**) FISH assay verifying the overexpression of SNORD72. (**F**) CCK-8 assay showing overexpression of SNORD72 increases the proliferation ability compared with control group *in vitro*. (**G**) Colony formation assay showing overexpression of SNORD72 increases the growth ability compared with control group *in vitro*. (**H**) Transwell invasion assay showing overexpression of SNORD72 increases the invasion ability compared with control group *in vitro*. (**I**) qRT-PCR assay showing the effect of SNORD72 on the expression of ID2. (**J**) Western blot assay showing the effect of SNORD72 on the expression of ID2. (**K**) FISH assay showing the effect of SNORD72 on the expression of ID2. (**L**) The effect of SNORD72 on the mRNA stability of ID2. *: P < 0.05; **: P < 0.01; ***: P < 0.001.

Some members of the SNORD family have been implicated in the pathological process of cancer biology [6]. But the role of SNORD72 in cancer has not been elucidated. Firstly, the expression of snoRNAs which were affected by lncRNA-LALR1 in HCC tissues was analysed by using the snoRNA database (http://bioinfo.life.hust.edu.cn/SNORic). We found that SNORD72 was highly expressed in HCC tissues in comparison to normal liver tissues (*P*<0.001) (Figure 6A). SNORD51 was highly expressed, but the expression of SNORA20, SNORD10 and SNORD14D was significantly reduced in HCC tissues (*P*<0.05) (Supplementary Figure 5A–5D). We then validated that SNORD72 was highly expressed in HCC samples by qRT-PCR (*P*<0.05) (Figure 6B). Furthermore, SNORD72 was highly expressed in samples with poor differentiation (*P*<0.01) (Figure 6C). Other snoRNAs have less if any correlation with clinicopathological parameters (Supplementary Figure 5E–5H). We then overexpressed SNORD72 in SMMC-7721 cells (Figure 6D–6E) to determine the biological role of SNORD72 in HCC. We found that overexpression of SNORD72 significantly promoted HCC cells proliferation (*P*<0.001) (Figure 6F). Consistently, overexpression of SNORD72 led to enhanced colony formation capacity of HCC cells (*P*<0.01) (Figure 6G). Moreover, SNORD72 also increased the invasion capcacity of HCC cells (*P*<0.001) (Figure 6H). Meanwhile, overexpression of SNORD72 was found to increase the expression of ID2 (*P*<0.05) (Figure 6I–6K) and stabilize ID2 mRNA (*P*<0.01) (Figure 6L). We also found that SNORD72 did not affect the expression and stability of lncRNA-LALR1 (Supplementary Figure 6A–6B). SnoRNAs can regulate ribosome biogenesis [6]. However, we found that knock-down of lncRNA-LALR1 did not affect the biogenesis of ribosomes by detecting the expression of 18S and 28S (Supplementary Figure 7A–7D).

**Figure 7:**
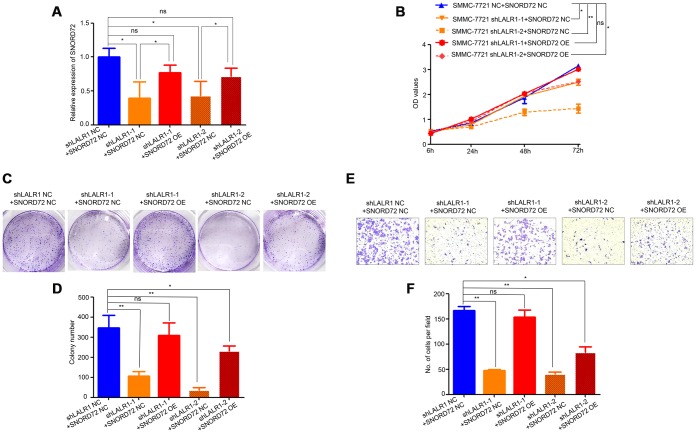
**Overexpression of SNORD72 restored the proliferation, colony formation and invasion capacity in lncRNA-LALR1-knockdown cells.** (**A**) qRT-PCR assay showing the overexpression of SNORD72 in lncRNA-LALR1-knockdown cells. (**B**) CCK-8 assay showing overexpression of SNORD72 rescues the proliferation ability compared with control group *in vitro*. (**C**) Colony formation assay showing overexpression of SNORD72 rescues the growth ability compared with control group *in vitro*. (**D**) Colony formation assay showing overexpression of SNORD72 rescues the growth ability compared with control group. (**E**) Transwell invasion assay showing overexpression of SNORD72 rescues the invasion ability compared with control group *in vitro*. (**F**) Transwell invasion assay showing overexpression of SNORD72 rescues the invasion ability compared with control group. *: P < 0.05; **: P < 0.01; ***: P < 0.001.

We then overexpressed SNORD72 in lncRNA-LALR1-knockdown cells and discovered SNORD72 expression also increased compared with corresponding negative control cells. (*P*<0.05) (Figure 7A). We found that overexpression of SNORD72 restored the proliferation (Figure 7B) and colony formation (Figure 7C–7D) of SMMC-7721 cells. Moreover, overexpression of SNORD72 also increased the invasion capacity of lncRNA-LALR1-knockdown cells (Figure 7E–7F).

These results demonstrated that SNORD72 is highly expressed in HCC tissues mediates the pro-tumor effect of lncRNA-LALR1 in HCC via stabilzing ID2 mRNA.

## DISCUSSION

Although great progress has been made in the diagnosis and treatment of HCC in the past few decades, the prognosis of HCC patients is still poor. It is urgent to better understand the pathological and molecular mechanism and provide novel targets for the diagnosis and treatment of HCC. Recent studies demonstrated some lncRNAs were deregulated in HCC specimen, and associated with the clinicopathological features of HCC. These lncRNAs modulate cell proliferation, apoptosis, autophagy, epithelial-mesenchymal transition (EMT), invasion and metastasis of HCC through regulating gene expression and cancer-related signaling pathways [3]. Previous work by Xu Dan and colleagues revealed that lncRNA-LALR1 promoted hepatocytes proliferation by promoting cell cycle in mouse liver regeneration [4], but its role was not clarified in HCC. Our present study found that lncRNA-LALR1 was highly expressed in HCC tissues, and high expression of lncRNA-LALR1 was associated with advanced TNM stage, poor differentiation, and distant metastasis of the HCC. Moreover, we demonstrated that lncRNA-LALR1 promoted growth and invasion of HCC cells *in vitro* and *in vivo*.

Various mechanisms have been involved in the lncRNA-mediated gene regulation, which can be ascribed to their ability to interact with DNA, RNA or protein [17–19]. LncRNA regulates gene expression at transcriptional and post-transcriptional level. At the transcriptional level, lncRNA can influence gene transcription by directly binding to transcriptional complexes or regulating chromatin structure or DNA components such as promoter regions. At the post-transcriptional level, lncRNA can not only affect the mRNA stability, splicing and modification but also impact protein stability, mRNA translation and subcellular localization. Interestingly, we found that lncRNA-LALR1 was expressed not only in the cytoplasm, but also in nucleus. Moreover, knockdown of lncRNA-LALR1 affected the expression of some snoRNAs including SNORD72. Most recently, lncRNA AY was demonstrated to be highly expressed in HCC tissues and expressed in both cytoplasm and nucleus [20]. Nuclear lncRNA AY interacted with histone 1FX (H1FX) to promote the transcription of integrin αV. Another nuclear lncRNA called SCARNA10 was also reported to bind with polycomb repressive complex 2 (PRC2) to prevent its binding to the promoters of target genes [21]. In the present study, we showed that lncRNA-LALR1 could up-regulate and bind with SNORD72. However, knock-down of lncRNA-LALR1 did not affect the stability of SNORD72. We can speculate that lncRNA-LALR1 might regulate the transcription or maturation of SNORD72. Although most snoRNAs in human are produced from protein-coding genes by splicing [22], we found that the SNORD72 gene was located far away from other genes (Supplementary Figure 7E), implying that it can be produced in an independent way. In what manner SNORD72 expression is regulated should be revealed in the future work.

We further demonstrated that SNORD72 was highly expressed in HCC tissues and SNORD72 increased the proliferation and invasion of HCC cells. Consistently, overexpression of SNORD72 remarkably increased the expression of ID2. The downstream signaling of snoRNAs is largely unknown. Although it was previously reported that the snoRNAs regulated ribosome biogenesis [5], a large subclass of snoRNAs were orphan snoRNAs which did not match any other RNA sequence [22]. We found that ribosome biogenesis was not affected after overexpression of SNORD72 (Supplementary Figure 6C–6D), implying it exerts functionality in other ways. On one hand, snoRNAs could regulate the expression of RNAs. For example, Yu-Hang Xing and colleagues reported that SNORAs could bind and stabilize lncRNA SLERT to modulate RNA helicase DDX21 to regulate the expression of rRNAs [23]. SNORD83B could reduce the stability of NOP14, SRSF3, and RPS5 [24]. On the other hand, snoRNAs also directly activate or suppress enzymes. It has been reported that SNORD50A and SNORD50B bind with K-RAS to inhibit its activity [25]. Some SNORAs could activate directly PARP-1 to promote ribosome biogenesis [26]. As previously reported ID2 could not only promote the proliferation and inhibit apoptosis of HCC cells [13, 14], but also induce invasion and metastasis of HCC cells [15]. We found that both lncRNA-LALR1 and SNORD72 could increase the stability of ID2 mRNA. Most recently, lncRNA ANCR was reported to stabilize ID2 mRNA by interacting with PTBP1 [27].

In conclusion, we reveal that lncRNA-LALR1 is highly expressed in HCC samples and correlates with advanced tumor stage. LncRNA-LALR1 promotes proliferation and invasion of HCC cells by up-regulating SNORD72 to stabilize ID2 mRNA. SNORD72 is overexpressed in HCC tissues and enhances cell proliferation and invasion. These results suggest that lncRNA-LALR1 might be a novel target for the diagnosis and treatment of HCC.

## MATERIALS AND METHODS

### Human specimens and cell culture

The HCC tumor tissues and paired adjacent tissues were acquired from 46 patients who underwent surgery at the 2^nd^ Affiliated Hospital of Chongqing Medical University (Chongqing, China) between 2016 and 2017, with the approval of the Institutional Review Board of Chongqing Medical University. Patients provided informed consent and had not received chemotherapy or radiation therapy before surgery. All liver specimens were immediately collected after surgery and stored at liquid nitrogen for further use. The human HCC cell line Huh7, HepG2, SMMC-7721, SK-Hep1, PLC/PRF/5, MHCC-97H were obtained from the American Type Culture Collection. All cells in the experiments were maintained in Dulbecco's Modified Eagle Medium (HyClone, Logan, UT, USA). All culture media were supplemented with 10% FBS (Gibco, Rockville, MD, USA),100 units/mL penicillin, and 100 μg/mL streptomycin (HyClone) in a humidified incubator of 5% CO_2_ and 95% air at 37 °C.

### RNA fluorescence in situ hybridization analysis (FISH)

Cells were denatured by a series of chemical reagents. Then hybridize with a predenaturing FISH probe overnight. The next day washed with buffer solution and stained with DAPI for detection according fluorescent in situ hybridization kit manufacturer (Genepharma, Shanghai, China). Further details are available in Supplementary Methods.

### RNA sequencing (RNA-Seq)

RNA Sequencing was accomplished by Sangon Biotech (Shanghai, China). Total RNAs were isolated from cells using Trizol reagent (Invitrogen, Carlsbad, CA, USA) and quantified by Nanodrop 2000 spectrophotometer and Qubit 3.0 fluorometer (Thermo Scientific, USA). Their quality was also evaluated using Agilent 2100 Bioanalyzer and all RNA samples had RNA integrity numbers ≥8. Total RNA was subsequently used to prepare RNA-seq libraries using TruSeq RNA Sample Pre Kit v2 (Illumina), Agencourt ® AMPure XP Beads (Beckman), SuperScript II Reverse Transcriptase (Invitrogen, Carlsbad, CA, USA), Qubit™ dsDNA HS Assay Kit(Invitrogen, Carlsbad, CA, USA)and Qubit™ dsDNA HS Assay Kit (Invitrogen, Carlsbad, CA, USA) following the manufacturers’ protocols. The libraries were paired-end sequenced with HiSeq 2500 (Illumina). The differentially expressed mRNAs were identified using GeneSpring software version 13.0 (Agilent, CA) after expression data of mRNAs were filtered and normalized. The functional enrichment analysis was carried out using the KEGG Orthology Based Annotation System (KOBASS).

### RNA-RNA pulldown assay

LncRNA-LALR1 denatured biotin-labeled RNA probes were designed and synthesized by Genepharma (Shanghai, China). RNAs were extracted from cells and purified. The denatured biotin-labeled RNA probe was incubated with prewashed Streptavidin Magnetic Beads (Thermo Scientific, USA) for 30 min at room temperature with agitation. After washing the beads, equal amounts of transcripts were hybridized with the beads in LiCl hybridization solution at 37°C for 2 hours. The binding components were pulled down by biotin-labeled RNA probes and analyzed by qRT-PCR [28].

### Lentivirus infection

Lentiviruses carrying small hairpin RNA (shRNA) sequence of human lncRNA-LALR1 were synthesized by Obio Company (Shanghai, China). Cells were planted into six-well plates and cultured overnight. They were washed with PBS and 2mL serum free medium was added per well. Lentivirus was added directly to the medium (MOI=20). Twenty-four hours later, the medium containing the lentivirus was discarded and replaced with new medium. After 72 h, puromycin was added to the medium for one week to select stably infected cells. Finally, total RNA were extracted from the stably infected cells to assess the efficiency of knockdown.

### Cell proliferation assay

Cells were plated in 96-well plates at a density of 5×10^3^ cells/well and measured using Cell Counting Kit-8 (CCK-8) (Dojindo Laboratories, Kumamoto, Japan). Cell proliferation was determined every 24 h for three days following the manufacturer’s protocol. The optical density was measured with a microplate reader (Bio-Rad, Hercules, CA, USA). Triplicate independent experiments were performed.

### Colony formation assay

For the colony formation assay, 1.0×10^3^ cells were seeded in a six-well plate. After 15 days, colonies were fixed and stained with 0.1% crystal violate (Invitrogen, Carlsbad, CA, USA) before counting. Triplicate independent experiments were performed.

### Cell invasion assay

The invasion of SMMC-7721 and HepG2 cells was evaluated in transwells (BD Falcon, Franklin Lakes, NJ). Briefly, 2×10^4^ cells were seeded into Matrigel^TM^ (BD Biosciences, Franklin Lakes, NJ)-coated upper chambers. After incubation for 48 hours at 37°C, the remaining cells left in the upper chamber were removed and the cells in the lower chambers were fixed with 4% polyformaldehyde and stained with 0.1% crystal violet. The number of cells was counted in 5 distinct areas at ×100 magnification. The results represent the average cell number in 3 wells per cell line.

### Cell cycle analysis

Cells were harvested at a density of 1.0×10^6^ cells/mL, washed with PBS, and fixed with 75% ethanol at 4 °C overnight. Then, the cells were washed with PBS, stained with propidium iodide, and performed to cell cycle analysis using a FACSCalibur instrument (BD Biosciences, Franklin Lakes, NJ, USA) and CellQuest software [29].

### Cell apoptosis assay

Cells were obtained at a density of 1.0×10^6^ cells/mL and incubated with reagents from the Annexin-V-FITC Apoptosis Detection Kit (Neobioscience, Shenzhen, China) according to the manufacturer’s protocol. Then the cells were analyzed by FACS Vantage SE flow cytometer (BD Biosciences, San Jose, CA, USA). The experiment was triplicated repeated independently.

### mRNA stability

After the cells were seeded in 6-well plates overnight, and then treated with actinomycin D(ActD) (MedChemExpress, USA), which was added to inhibit further RNA synthesis at a final concentration of 5μg/mL. The cells were treated with different time intervals in the presence of actinomycin D (0, 2, 4, 8, 12 or 24 hours). Total RNA was extracted and analyzed by qRT-PCR. The remaining RNA levels of interest at each time point were normalized to that at the beginning (0 hours).

### Subcutaneous tumor transplantation in nude mice

The use and care of animals was approved by the Institutional Animal Care and Use Committee at Chongqing Medical University. To evaluate the in vivo tumorigenic effects, 5×10^5^ SMMC-7721 cells were suspended in serum-free-DMEM/Matrigel mixture (1:1 volume) and injected subcutaneously into the flank of nude mice. Tumor volume was monitored by measuring the length and width using vernier calipers at 7-day intervals, for consecutive 5 weeks. After 5 weeks, mice were euthanized, and xenografts were harvested and weighed. The tumor tissues were dissected out and fixed with formalin for pathological examination.

### Statistical analysis

All values were presented as means ± standard deviation (SD). All data were obtained from at least three repetitions of each experiment. And the statistical significance between treatment and control groups was analyzed with GraphPad Prism 5.0 software (La Jolla, CA, USA). Statistical significance was determined using ANOVA for multiple comparisons and Student’s t-test was used to compare two groups. Correlations between lncRNA-LALR1 expression and individual clinicopathologic parameters were evaluated using a nonparametric χ^2^ test. Probability values (p) less than 0.05 was considered statistically significant.

## Supplementary Material

Supplementary Figures
