# Supplementary material for: LncRNA-LALR1 upregulates small nucleolar RNA SNORD72 to promote growth and invasion of hepatocellular carcinoma
Source: Aging (Albany NY). 2020 Mar 11;12(5):4527–46. doi: 10.18632/aging.102907 (PMC7093170; doi:10.18632/aging.102907)
Supplement: Supplementary Figures [file aging-12-102907-s001..pdf]

SUPPLEMENTARY FIGURES

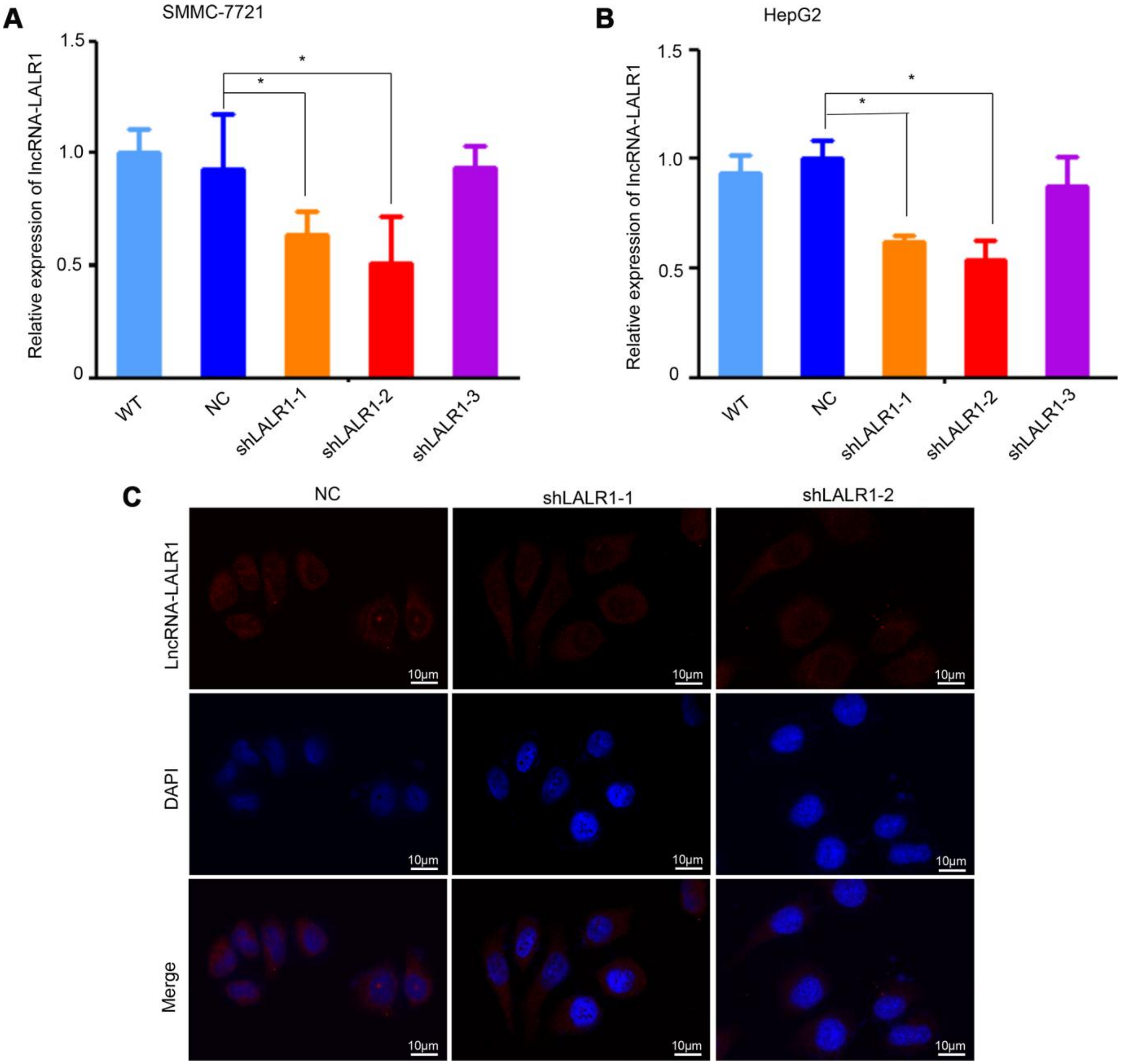

Supplementary Figure 1. Validation of knock-down of lncRNA-LALR1 in HCC cells. (**A**) qRT-PCR assay verifying the knock-down of lncRNA-LALR1 in SMMC-7721 cells. (**B**) qRT-PCR assay verifying the knock-down of lncRNA-LALR1 in HepG2 cells. (**C**) FISH assay verifying the knock-down of lncRNA-LALR1 in SMMC-7721 cells. **: P < 0.05; **: P < 0.01; ***: P < 0.001; ns: no significant.*

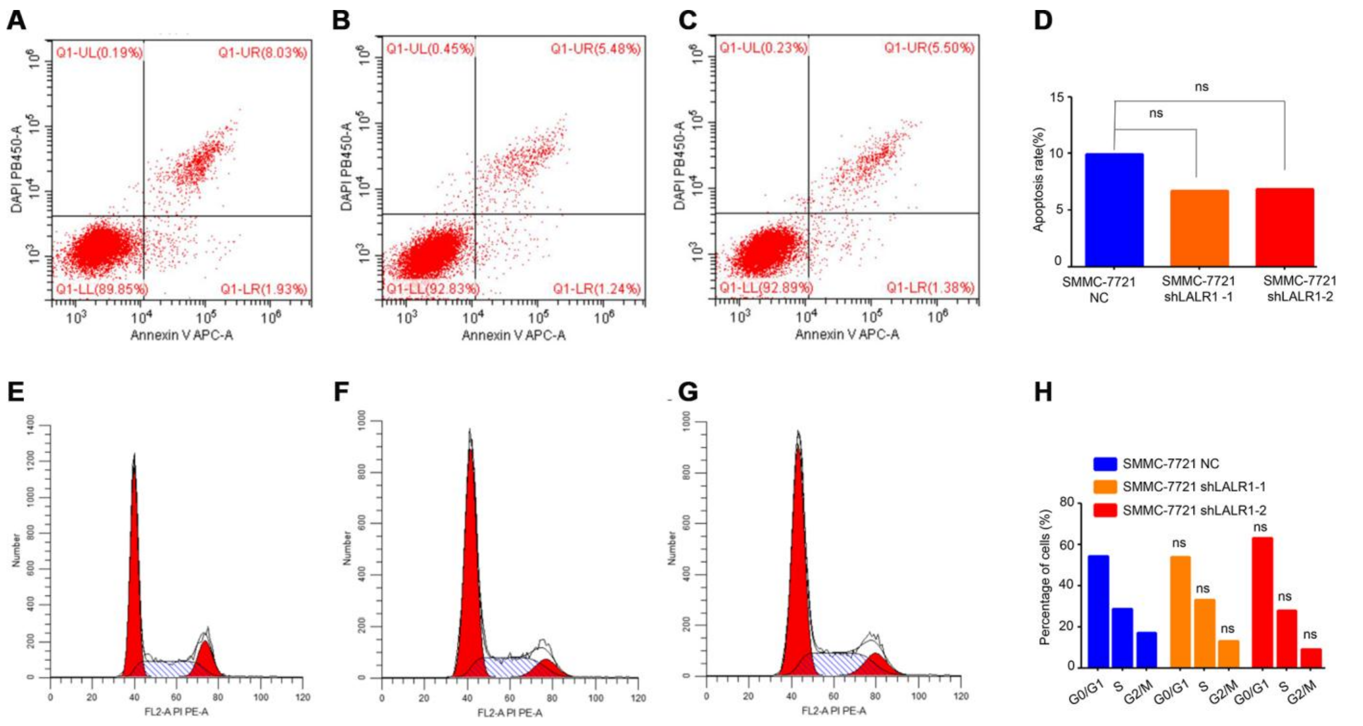

Supplementary Figure 2. Knock-down of lncRNA-LALR1 has no effect on apoptosis and cell cycle of HCC cells. (**A**) The effect of control group on apoptosis of SMMC-7721 cells. (**B**) The effect of lncRNA-LALR1-knockdown-1 cells on apoptosis. (**C**) The effect of lncRNA-LALR1-knockdown-2 cells on apoptosis. (**D**) Knock-down of lncRNA-LALR1 has no effect on apoptosis of HCC cells. (**E**) The effect of control group on cell cycle of SMMC-7721 cells. (**F**) The effect of lncRNA-LALR1-knockdown-1 cells on cell cycle. (**G**) The effect of lncRNA-LALR1-knockdown-2 cells on cell cycle. (**H**) Knock-down of lncRNA-LALR1 has no effect on cell cycle of HCC cells. *ns: no significant.*

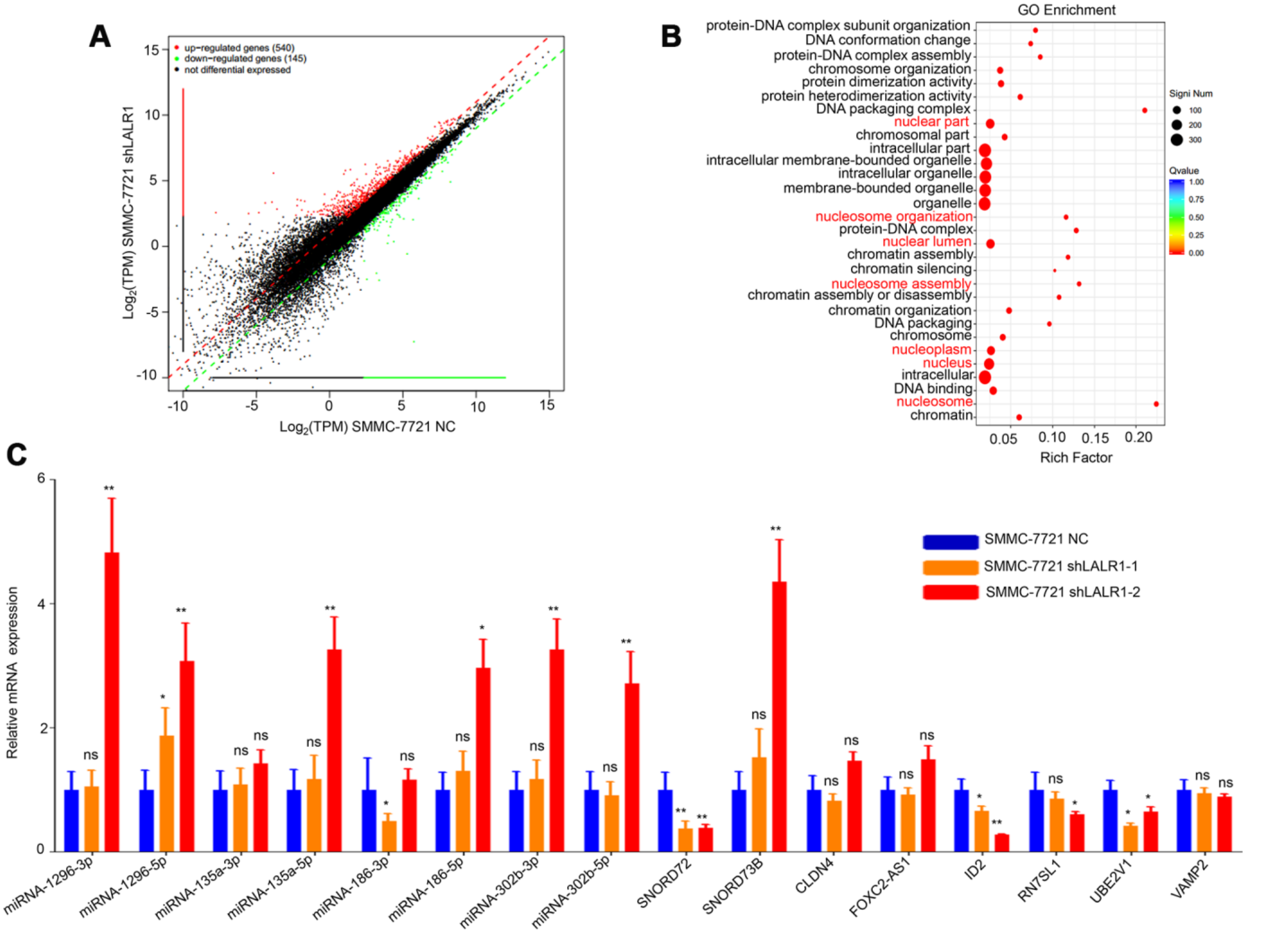

Supplementary Figure 3. Differentially expressed genes after knock-down of lnc-LALR1 in HCC cells. (**A**) Knockdown of lncRNA-LALR1 upregulates 540 genes and downregulates 145 genes (fold change>2,P <0.05). (**B**) GO analysis reveales that lncRNA-LALR1 was mainly related with nuclear components and processes. (**C**) qRT-PCR assay showing the expression of the top 16 differentially expressed genes expression and showing that knockdown of lncRNA-LALR1 significantly decreased SNORD72 and ID2 expression. **: P < 0.05; **: P < 0.01; ***: P < 0.001. ns: no significant.*

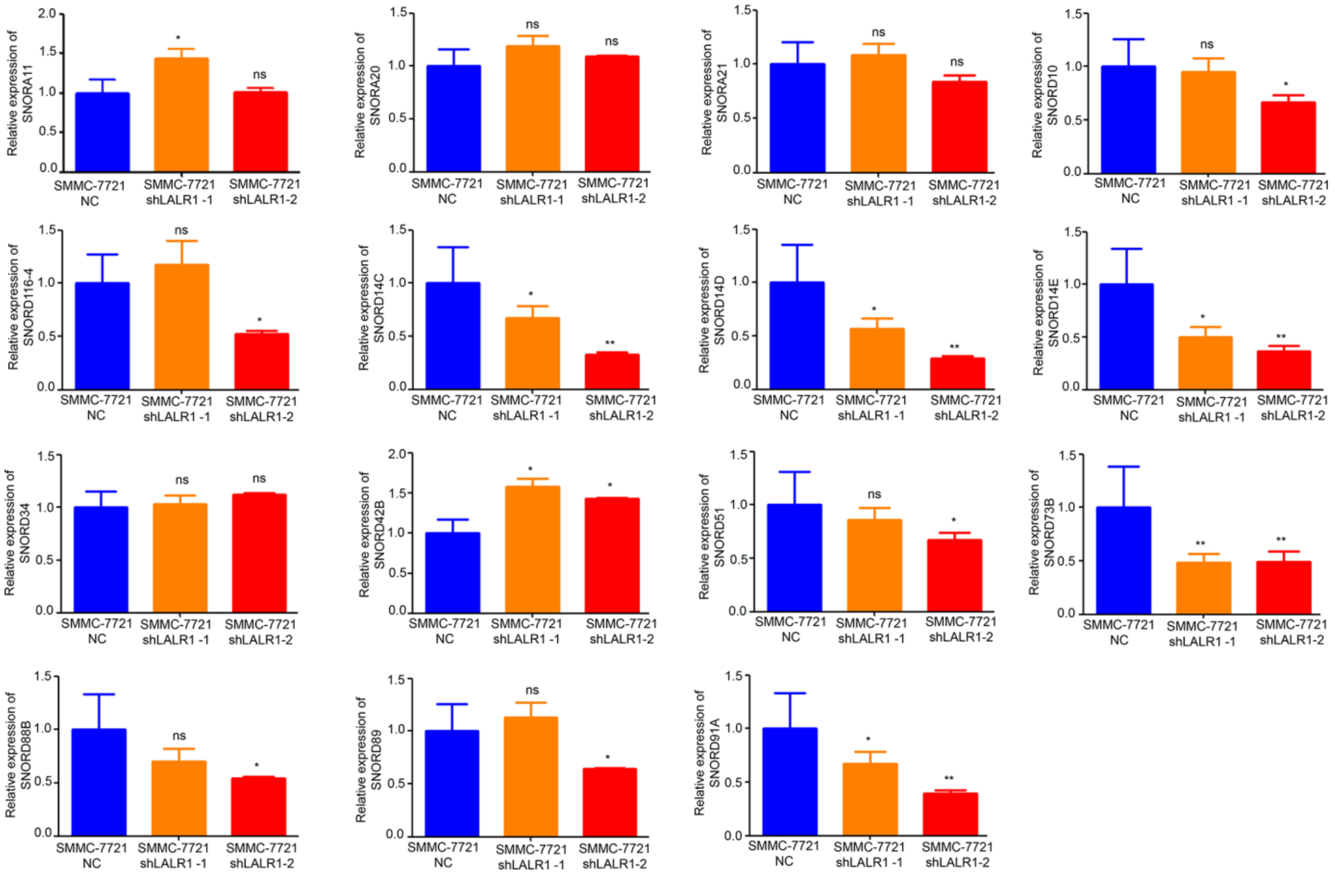

Supplementary Figure 4. Knock-down of lncRNA-LALR1 changed the expression of other snoRNAs to a lesser degree.

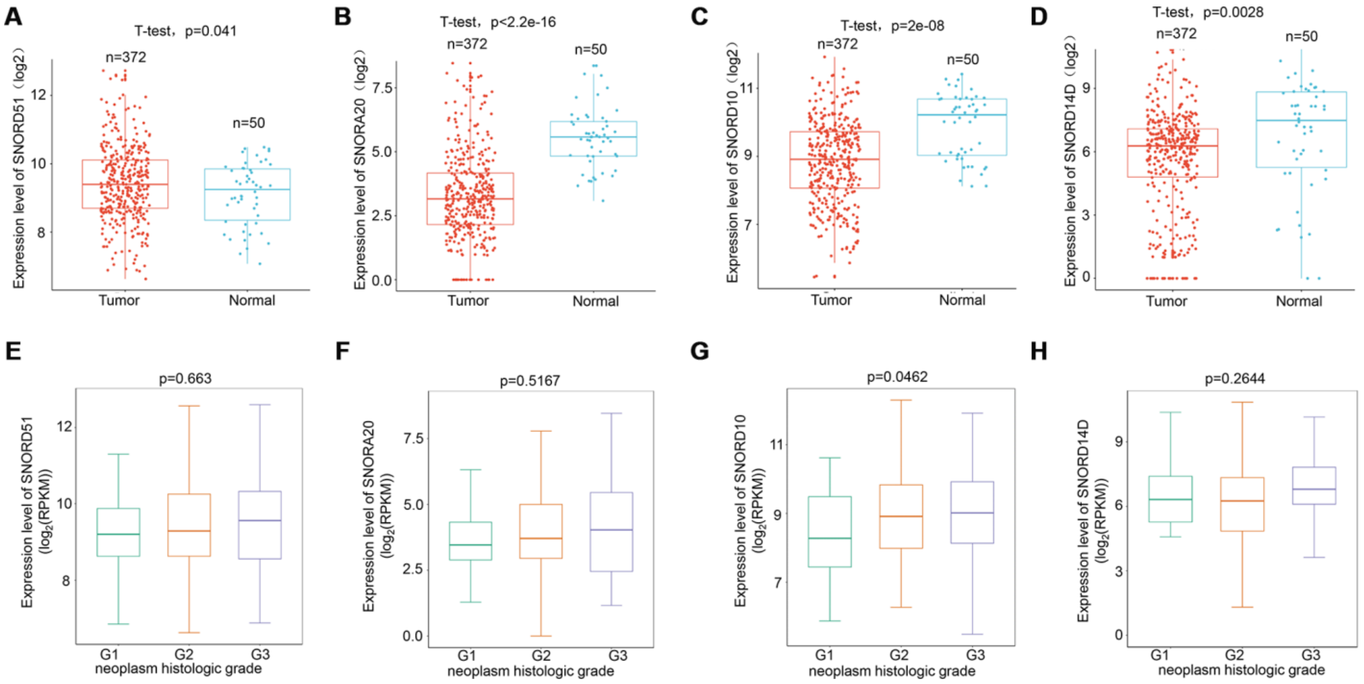

Supplementary Figure 5. The expression and differentiation of other snoRNAs in HCC. (**A**) Bioinformatic analysis showing the expression of SNORD51 in HCC tissues. (**B**) Bioinformatic analysis showing the expression of SNORA20 in HCC tissues. (**C**) Bioinformatic analysis showing the expression of SNORD10 in HCC tissues. (**D**) Bioinformatic analysis showing the expression of SNORD14D in HCC tissues. (**E**) The differentiation of SNORD51 in HCC. (**F**) The differentiation of SNORA20 in HCC. (**G**) The differentiation of SNORD10 in HCC. (**H**) The differentiation of SNORD14D in HCC. **: P < 0.05; **: P < 0.01; ***: P < 0.001*.

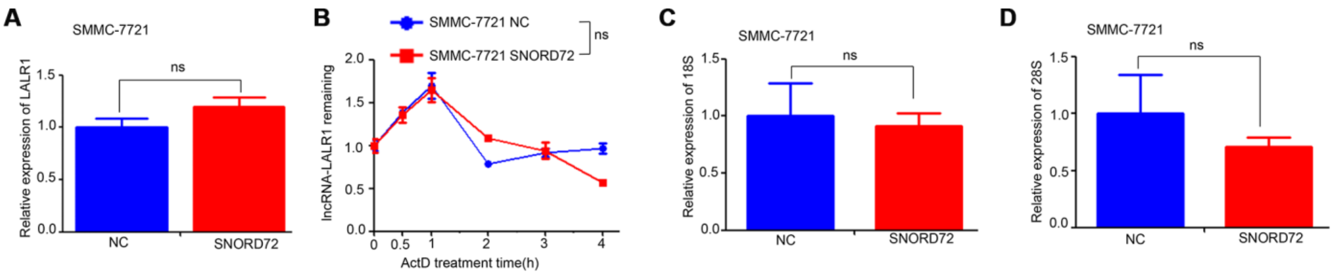

Supplementary Figure 6. Overexpression of SNORD72 did not affect the expression and stability of lncRNA-LALR1 and ribosome biogenesis. (**A**) qRT-PCR assay showing the effect of SNORD72 overexpressed on the expression of lncRNA-LALR1. (**B**) The effect of SNORD72 on the mRNA stability of lncRNA-LALR1. (**C**) qRT-PCR assay showing the effect of SNORD72 overexpressed on the expression of 18S. (**D**) qRT-PCR assay showing the effect of SNORD72 overexpressed on the expression of 28S in SMMC-7721 cells. *ns: no significant.*

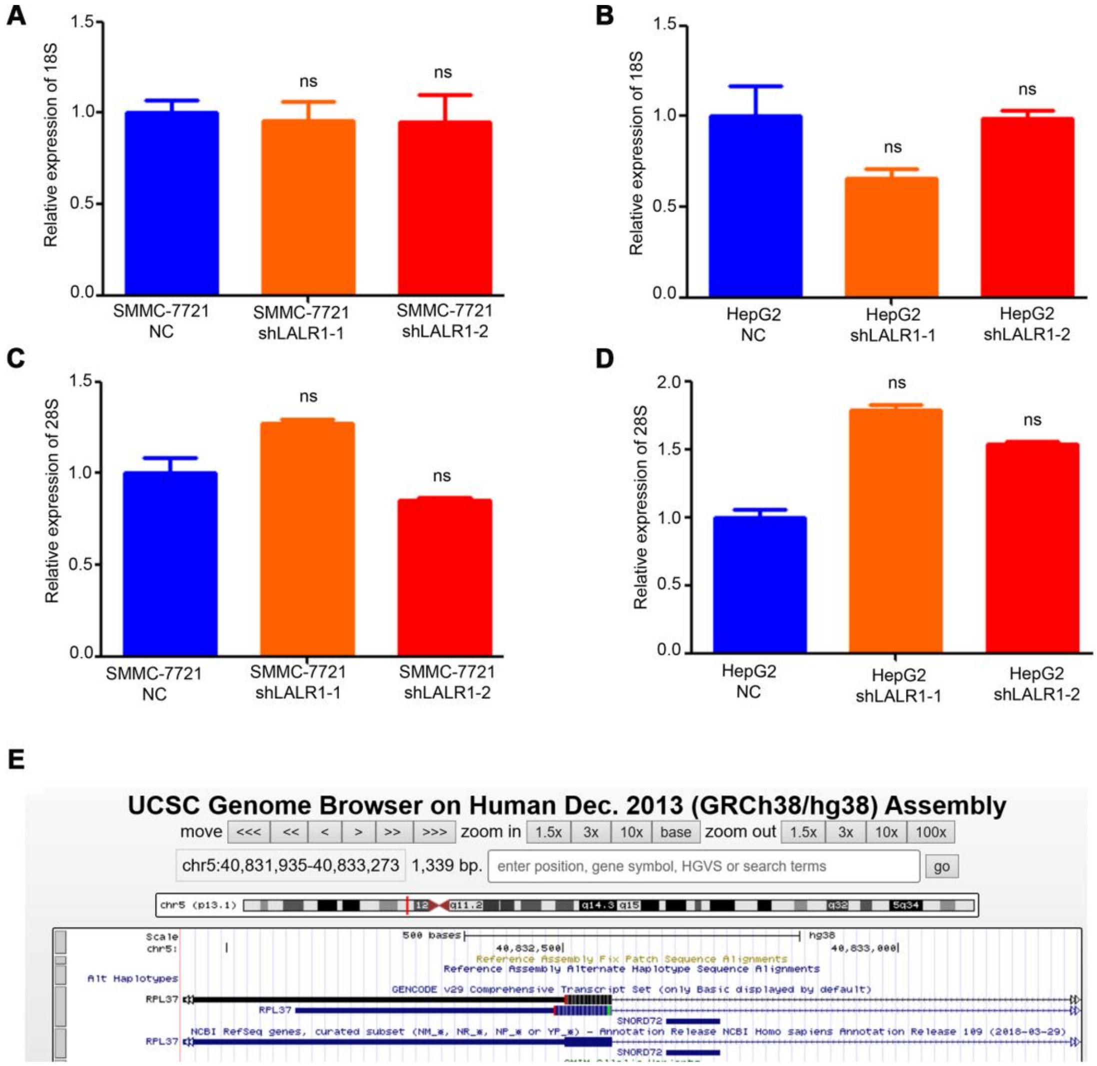

Supplementary Figure 7. LncRNA-LALR1 does not affect the biogenesis of ribosomes. (**A**) qRT-PCR assay showing the effect of knockdown lncRNA-LALR1 on the expression of 18S in SMMC-7721 cells. (**B**) qRT-PCR assay showing the effect of lncRNA-LALR1 silencing on the expression of 18S in HepG2 cells. (**C**) The effect of knockdown lncRNA-LALR1 on the expression of 28S in SMMC-7721 cells. (**D**) The effect of lncRNA-LALR1 silencing on the expression of 28S in HepG2 cells. (**E**) Public database showing the location of SNORD72 in chromosome. *ns: no significant.*
